# Correlation between depression and intimacy in lung cancer patients and their family caregivers

**DOI:** 10.1186/s12904-022-00992-7

**Published:** 2022-06-03

**Authors:** Chuanzhen Li, Juan Yuan, Xiaoxiao Huang, Siwen Zhang, Yutong Hong, Jiudi Zhong

**Affiliations:** Department of Thoracic Surgery, Sun Yat-Sen University Cancer Center, 651 Dongfeng East Road, Guangzhou, 510060 People’s Republic of China

**Keywords:** Lung cancer, Family caregivers, Depression, Intimacy, Actor-partner interdependence model

## Abstract

**Background:**

Cancer impacts both patients and their family caregivers. This study aimed to explore the interdependence between depression and intimacy in lung cancer patients and their family caregivers, providing the basis for developing a patient-caregiver centered dyadic intervention.

**Methods:**

This cross-sectional study recruited 182 dyads of lung cancer patients and their family caregivers using a convenient sampling. The depression subscale of the Hospital Anxiety and Depression Scale (HADS) and the Mutuality Scale (MS) were used to measure participants’ depression and intimacy respectively; and the correlation between depression and intimacy in patients and caregivers was analyzed by establishing the actor-partner interdependence model.

**Results:**

Thirty four percent of the patients and 19.2% of the caregivers were at risk of depression, with an intimacy score of 2.67 ± 0.74 points and 2.6 ± 0.86 points, respectively; Pearson correlation analysis showed that there was a positive correlation between the depression score (*r* = 0.226, *P* < 0.01) and intimacy score (*r* = 0.344, *P* < 0.01) in patients and caregivers; and the results of actor-partner interdependence model showed that caregivers’ depression had an actor effect on their own intimacy (b = -0.054, *P* = 0.004) as well as a partner effect on patients’ intimacy (b = -0.041, *P* = 0.011). However, patients’ depression has no influence on the intimacy of patients or caregivers.

**Conclusions:**

There is an interdependent relationship between depression and intimacy in lung cancer patients and family caregivers. Therefore, dyadic interventions can help them to cope with cancer together.

## Background

Lung cancer is a common malignant tumor that threatens human health. According to global cancer statistics, there were up to 2.2 million new cases of lung cancer and 1.8 million deaths in 2020 [1]. In China, the incidence and mortality rates of lung cancer are at the top of malignant tumors [2]. Due to the poor prognosis and low 5-year survival rate of lung cancer, patients have a higher incidence of depression, which seriously affects their quality of life [3, 4]. Cancer, as a dyadic stress, not only affects the physical and mental health of patients, but also puts tremendous physical, psychological, economic and social stress on family caregivers. With the heavy burden of caregiving, 22–32% of family caregivers may experience depression and a significant decrease in their quality of life [3, 5, 6]. During the diagnosis and treatment period, family caregivers, as the main carer and supporter of lung cancer patients, cope with cancer with patients together as a holistic "unit" [7].

In the Systemic Transactional Model (STM), Bodenmann [8] suggest that intimacy is an important protective factor for patients and family caregivers to cope with stressful events together and can effectively facilitate their adaptation to the disease. There is an interaction of patients’ and family caregivers’ intimacy through verbal or nonverbal means on the premise of mutual understanding, care, and trust, thus satisfying the need for love and belonging and generating psychological and physical closeness [9]. Some studies show that for diseases such as stroke, dementia and colorectal cancer, good intimacy can buffer the effect of stress on patients and family caregivers and play a significant moderating role between depression and their quality of life, helping them to go through the distressing experience associated with the disease [10–12]. Currently, domestic and international studies have focused primarily on the effect of lung cancer on the physical and mental health of patients or family caregivers, with insufficient attention paid to the patient-caregiver intimacy in coping with the disease, and no studies have explored the correlation between depression and intimacy at the dyadic level. In this study, we took lung cancer patients and family caregivers as dyads in the actor-partner interdependence model to investigate the effect of their depression on the intimacy between them, aiming to provide a theoretical basis for developing dyadic nursing interventions to promote their co-adaptation to the disease.

## Methods

### Participants

This cross-sectional study recruited 182 dyads of lung cancer patients and their family caregivers using a convenient sampling (364 participants in total). Patients were hospitalized and underwent surgical treatment at a specialized oncology hospital in Guangdong Province from March to April 2021. The inclusion criteria for patients are: (1) pathologically diagnosed as primary lung cancer; (2) having received surgical treatment; (3) age ≥ 18 years; (4) no cognitive and communication impairment; and (5) having obtained an informed consent. The exclusion criteria for patients are: (1) experiencing deterioration and serious complications after surgery; and (2) Patients under illness protection. The inclusion criteria for family caregivers are: (1) family members who spent most time caring for the patients, including parents, children, spouse, siblings, etc., or family caregivers designated by the patients; (2) age ≥ 18 years; (3) no cognitive and communication impairment; and (4) having obtained an informed consent. The exclusion criteria for family caregivers are being paid for taking care of patients.

According to the Kendall sample estimation method, the sample size should be approximately 5–10 times the number of the explanatory variables [13]. It was estimated that there were 17 explanatory variables in this study. Based on 10 times of the explanatory variables, a sample size of a 170 was calculated.

### Measures

#### General information questionnaire

Designed by the researcher, the general information questionnaire for patients includes age, gender, marriage, education, per capita monthly income, cancer pathological type, stage, with or without postoperative complications, etc. The general information questionnaire for family caregivers includes age, gender, marriage, education, per capita monthly income, relationship with patients, living with patients or not, etc.

#### Hospital Anxiety and Depression Scale (HADS)

HADS was developed by Zigmond and Snaith [14] to screen individuals for possible anxiety and depression disorders, and was translated into Chinese by Ye and Xu [15]. The scale has 2 subscales, anxiety subscale and depression subscale, with a total of 14 items. The depression subscale used in this study contains 7 items and is used to measure the depressive mood of individuals, and the Likert 4-level scale was used with the score range of 0–3 points and the total score range of 0–21 points. The higher the score is, the more severe the depression is. 0–7 indicates no depression, 8–10 indicates suspected depression, and 11–21 indicates depressive symptoms. The depression subscale has good reliability and validity, and the Cronbach's alpha coefficient is 0.806.

#### Mutuality Scale (MS)

MS was invented by Archbold et al. [16] to explore the intimacy between patients and caregivers, and the Chinese version was translated and verified by Xu et al. [17]. The scale includes 4 dimensions of love, shared joy, shared values and reciprocity, with a total of 15 items. The Likert 5-level scale was adopted with 0 indicating "not at all" and 4 indicating "very much", and the scale was scored on an average basis, with higher scores indicating better intimacy. The scale has been proved to have good reliability and validity, with Cronbach's alpha coefficient of 0.91.

### Data collection

This study was approved by the cancer center’s Institutional Review Board. After obtaining written informed consent from patients and family caregivers, trained investigators introduced the purpose of the survey to the participants and explained the method of completing the questionnaire using the uniform instructions. Patients and family caregivers were required to complete the questionnaire separately and independently, or if they were unable to do the survey on their own, the participants could dictate their answers and the investigators could help them fill the questionnaires. The questionnaires were distributed and collected on site, and the investigators checked the collected questionnaires and filled in blank items on a timely basis. A total of 364 questionnaires were distributed, and 364 valid questionnaires were collected, with a valid response rate of 100%.

### Statistical analysis

SPSS 25.0 statistics software was used for data analysis. Descriptive statistics were used for general information; paired t-test was used to compare the depression and intimacy scores of patients and family caregivers; Pearson correlation analysis was used to analyze the correlation between depression and intimacy in patients and family caregivers; actor-partner interdependence model was used to analyze the effect of depression of patients and family caregivers on their intimacy with each other. The differences are statistically significant at *P* < 0.05.

Actor-partner interdependence model is a method for analyzing the correlation of dyadic data which can reduce the probability of making Type I and II errors when analyzing two sets of non-independent data compared with the conventional analysis method [18]. Actor-partner interdependence model can not only analyze the effect of one's own prediction variable X (e.g., patient depression) on his/her outcome variable Y (e.g., patient intimacy), which is referred to as the actor effect, but also the effect on his/her spouse's outcome variable Y (e.g., family caregiver intimacy), which is referred to as the partner effect. After creating a paired dataset of lung cancer patients and family caregivers, the data was centrally preprocessed and a two-intercept model was established through a multilevel modeling approach to analyze the actor and partner effects of patients and family caregivers.

## Results

### Characteristics of lung cancer patients and family caregivers

The lung cancer patients were 56.14 ± 11.18 years old, and the family caregivers were 45.73 ± 13.41 years old. Other basic information was shown in Table 1.

**Table 1:** Characteristics of lung cancer patients and family caregivers (*n* = 182 dyads)

Characteristics		Patients (%)	Caregivers (%)
Gender	Male	99 (54.4%)	95 (52.2%)
	Female	83 (45.6%)	87 (47.8%)
Marriage	Unmarried	7 (3.8%)	19 (10.4%)
	Married	164 (90.1%)	156 (85.7%)
	Divorced	7 (3.8%)	5 (2.7%)
	Widowed	4 (2.2%)	2 (1.1%)
Education	Elementary school and below	33 (18.1%)	18 (9.9%)
	Junior high school	52 (28.6%)	46 (25.3%)
	High school or secondary school	47 (25.8%)	41 (22.5%)
	College and above	50 (27.5%)	77 (42.3%)
Per capita monthly income (RMB)	< 2,000	22 (12.1%)	17 (9.3%)
	2,001–5,000	84 (46.2%)	75 (41.2%)
	5,001–10,000	49 (26.9%)	53 (29.1%)
	> 10,000	27 (14.8%)	37 (20.3%)
Pathological type	Squamous carcinoma	23 (12.6%)	-
	Adenocarcinoma	5 (2.7%)	-
	Others	154 (84.6%)	-
Staging	Stage 0	29 (15.9%)	-
	Stage I	107 (58.8%)	-
	Stage II	35 (19.3%)	-
	Stage III	10 (5.5%)	-
	Stage IV	1 (0.5%)	-
Complications	Yes	11 (6%)	-
	None	172 (94%)	-
Relationship with patients	Spouse	-	74 (40.6%)
	Children	-	68 (37.4%)
	Others	-	40 (22%)
Living with patients	Yes	-	135 (74.2%)
	No	-	47 (25.8%)

“-” indicates no data available

### Comparison of the depression and intimacy scores of lung cancer patients and family caregivers

Lung cancer patients have higher depression scores than family caregivers, and the difference is statistically significant. Among which, there were 37 (20.3%) patients with suspected depression and 25 (13.7%) patients with diagnosed depression; and there were 25 (13.7%) caregivers with suspected depression and 10 (5.5%) caregivers with diagnosed depression. The intimacy scores of patients and family caregivers were both high, and their difference is not statistically significant, as shown in Table 2.

**Table 2:** Comparison of the depression and intimacy scores of lung cancer patients and family caregivers

Variables	Patients (Mean ± SD)	Caregivers (Mean ± SD)	*t*	*P*
Depression	5.72 ± 4	4.26 ± 3.48	4.22	< 0.01
Intimacy	2.67 ± 0.74	2.6 ± 0.86	0.962	0.337

### Pearson correlation analysis of depression and intimacy in lung cancer patients and family caregivers

The depression and intimacy scores of lung cancer patients were positively correlated with those of family caregivers, respectively. Caregiver depression was negatively correlated with patient intimacy and caregiver intimacy; both the correlations are statistically significant, as shown in Table 3.

**Table 3:** Pearson correlation analysis of depression and intimacy in lung cancer patients and family caregivers

Variables	Patient depression	Patient intimacy	Caregiver depression
Patient depression	-		
Patient intimacy	-0.124	-	
Caregiver depression	0.226**	-0.211**	-
Caregiver intimacy	-0.099	0.344**	-0.23**

“**” indicates *P* < 0.01

### Actor-partner interdependence model analysis of depression and intimacy in lung cancer patients and family caregivers

The actor effect of family caregiver depression on their own intimacy is statistically significant (b = -0.054, *P* = 0.004), and the higher the level of caregiver depression is, the worse the caregivers' perceived intimacy with patients is. The partner effect of caregiver depression on patient intimacy is statistically significant (b = -0.041,*P* = 0.011), and the higher the level of caregiver depression is, the worse the patients’ perceived intimacy with caregivers is, as shown in Table 4.

**Table 4:** Actor-partner interdependence model analysis of depression and intimacy in lung cancer patients and family caregivers

Variables	*b*	*SE*	*P*
**Actor effect**			
Patient depression → Patient intimacy	-0.015	0.014	0.282
Caregiver depression → Caregiver intimacy	-0.054	0.018	0.004**
**Partner effect**			
Patient depression → Caregiver intimacy	-0.011	0.016	0.508
Caregiver depression → Patient intimacy	-0.041	0.016	0.011*

“*” indicates *P* < 0.05, “**” indicates *P* < 0.01

## Discussion

### Depression of lung cancer patients and family caregivers

Lung cancer is a major negative life event that not only causes patients to experience tremendous physical and psychological pain, but also puts heavy pressure on family caregivers. Both patients and caregivers often experience emotional distress such as depression during diagnosis and treatment period [3, 4, 6]. Tan et al. [5] found that depressed subjects were identified in 39.5% of the lung cancer patient sample and 27.9% of the caregiver sample. This is consistent with the findings of Lee et al. [6], reporting both patients’ and caregivers’ mean depression scores of HADS were over 6 point. In this study, the incidence of depression in lung cancer patients and their family caregivers were 34% and 19.2%, respectively, which are slightly lower than previous researches [4, 5]. The reason may be that most of the lung cancer patients in this study were in the early stages of cancer and had received surgery. Patients' symptoms, such as postoperative wound pain, weakened physical functions, and concerns about the prognosis of the disease, can all be the sources of patient depression [4, 19]. Family caregivers, as the patients’ main sources of care and support, often need to assist patients in disease management without proper training in addition to daily care. The long-term burden of caregiving may lead to various health problems such as pain, fatigue and poor sleep quality, accompanying with the concerns about the patient's prognosis and financial difficulties due to high treatment costs. Caregivers usually have a high incidence of depression [20, 21], which can sometimes even higher than that of patients [5]. In this study, lung cancer patients had higher levels of depression than family caregivers, possibly due to the fact that 59.4% of family caregivers were non-spouse caregivers. The findings of Siminoff et al. [22] showed that non-spouse caregivers have a lower depression score as compared to spouse caregivers.

### Intimacy of lung cancer patients and family caregivers

Lung cancer patients and family caregivers confront the challenges of cancer together as a whole unit, and the patient-caregiver intimacy is the cornerstone for them to cope with cancer together. This study shows that both patients and caregivers had a high perception of intimacy with each other, and there was no significant difference in the degree of intimacy between them, which was similar to the findings of Cai et al. [23]. Influenced by Confucian culture, Chinese people generally have a strong concept of family and blood kinship. The caregivers in this study were mostly spouses or children who lived with patient. The dyad had a good emotional foundation and could provide support and assistance to each other. A growing number of studies have confirmed that the intimacy of patients and family caregivers is significantly correlated with the depression and quality of life of both parties, and also is a major protective factor for facilitating them to effectively cope with the disease [10, 11, 23]. The findings of Pucciarelli et al. [11], which investigated stroke patients and caregivers [11], showed that on the one hand, the closer the patients’ intimacy with caregivers is, the higher their quality of life is; on the other hand, the caregivers’ perceived intimacy with the patients played a significant moderating role between depression and the quality of life, and intimacy can reduce the negative effect of depression on the caregivers’ quality of life. Therefore, maintaining a good intimacy between patients and family caregivers while coping with cancer together is conducive to improve the physical and mental health of both parties.

### The correlation between depression and intimacy in lung cancer patients and family caregivers

According to the systemic-transactional model [8], patients and family caregivers are an interdependent interactive system when facing the disease together, in which both parties mutually perceive, assess, communicate and cope with stress while getting along with each other. In this study, the depression of lung cancer patients was positively correlated with family caregivers’ depression, which is consistent with the findings of Tan et al. [5]. Caregivers who care for depressed patients often experience greater physical and psychological burden and a higher level of depression than those who care for patients without depression [5]. The findings also found that the intimacy of lung cancer patients was positively correlated with that of family caregivers, which is consistent with the findings of Luo et al. [24]. Regan et al. [25] have reported that during cancer diagnosis and treatment period, there is a change in the social and family roles of patients and caregivers, and they become interdependent with each other. When a party feels satisfied and happy with intimacy, the other party can also develop similar feelings. In addition, this study also shows that caregiver depression was significantly and negatively correlated with both caregiver intimacy and patient intimacy. This further confirms that patients and caregivers cope with cancer as a whole, rather than in separate. Therefore, patients and caregivers should be considered as a holistic "unit" when providing healthcare services to them, and each party should be taken as an important resource in their co-adaptation to the disease.

### Actor-partner interdependence model analysis of depression and intimacy in lung cancer patients and family caregivers

The actor-partner interdependence model analysis results show that only the depression of family caregivers has negatively actor and partner effects on the dyad’s intimacy, whereas patients’ depression has no influence on the intimacy of patients or caregivers. Similar to previous findings [11, 26], the higher the depression level of caregivers is, the worse their perceived intimacy is. In this study, caregiver depression not only influenced their own intimacy, but also impaired the intimacy of the patients who they are caring for. A possible reason maybe that the caregivers with higher level of depression are prone to adopt the negative coping behaviors, such as avoiding communication with the patients, blindly catering to the patients, leading to conflict between them; and in worse cases, some caregivers even take malignant caregiving behaviors such as patient abuse [5, 20]. The study of Luo et al. [24] on the gynecologic cancer couples found that the negative coping ways of the patient's spouse not only fail to eliminate bad feelings, but also destroy the couple's relationship, leading to a significant decrease of the couple’s intimate. Unlike caregivers, this study did not find significant actor and partner effects of lung cancer patients’ depression on the dyad’s intimacy. This may be due to the fact that patient depression is carefully managed by healthcare professionals and caregivers, and clinical mental healthcare services are mostly patient-centered, which could effectively alleviate patient depression and its adverse effect. However, family caregivers' mental health is often neglected and they also rarely seek for related health services, such as psychological counseling. Long-lasting depression not only increases caregivers' burden of caregiving, but also undermines the positive interaction between patients and caregivers, leading to a vicious circle [3, 5]. Therefore, in the future, healthcare professionals should also consider the needs of family caregivers when providing psychological problems screening and support services to patients. Notably, both the actor and partner effect of caregivers’ depression on the dyad’s intimacy found in this study are small. The most likely cause is the depression level of family caregivers in our study is low (the incidence of depression is only 19.2%), which might attenuate these effects.

This study has some limitations. First, as this is a cross-sectional study, we are unable to determine causality among the depression and intimacy of lung cancer patients and their family caregivers; therefore, longitudinal or interventional studies are needed. Second, we only explore the interdependent effects between the depression and intimacy of patients and caregivers. Considering the complexity of the interaction between the dyad, other variables, which might impact these associations (e.g. the dyad’ symptoms, self-efficacy), should be considered in future studies. Third, the participants were recruited from one tertiary hospital, which limits the generalizability of the current findings. We recommend future studies including more and different levels of hospitals to make the results more representative.

## Conclusions

In summary, when lung cancer patients and family caregivers coping with cancer together, the dyad interdepend on each other, and the depression and intimacy of patients are positively correlated with those of caregivers. In addition, the depressive mood of caregivers reduces not only their own perceived intimacy, but also that of the patients. The above findings suggest that healthcare professionals should treat patients and caregivers as a whole unit when providing healthcare services, paying attention to caregivers' psychological needs as well as patients. Dyadic nursing interventions, which centered on patients and caregivers, are warrant to develop to promote the mental health and intimacy of patients and caregivers, helping the dyad achieve co-adaptation to the disease.

## Data Availability

The data and materials are available from the corresponding author on reasonable request.
